# The GABA_A_ Receptor α2 Subunit Activates a Neuronal TLR4 Signal in the Ventral Tegmental Area that Regulates Alcohol and Nicotine Abuse

**DOI:** 10.3390/brainsci8040072

**Published:** 2018-04-21

**Authors:** Irina Balan, Kaitlin T. Warnock, Adam Puche, Marjorie C. Gondre-Lewis, Harry June, Laure Aurelian

**Affiliations:** 1 Department of Pharmacology, University of Maryland School of Medicine, Baltimore, MD 21201, USA; ibalan@som.umaryland.edu; 2 Neuropsychopharmacology Laboratory, Department of Psychiatry and Behavioral Sciences, Howard University College of Medicine, Washington, DC 20059, USA; kaitlin.warnock@gmail.com (K.T.W.); mgondre-lewis@Howard.edu (M.C.G.); harry.june@Howard.edu (H.J.); 3 Department of Anatomy and Neurobiology, University of Maryland School of Medicine, Baltimore, MD 21201, USA; apuche@som.umaryland.edu; 4 Laboratory for Neurodevelopment, Department of Anatomy, Howard University College of Medicine, Washington, DC 20059, USA; mgondre-lewis@Howard.edu

**Keywords:** TLR4 signal, PKA/CREB, CRF/TH, GABA_A_ α2, HSV siRNA vectors, alcohol/nicotine abuse

## Abstract

Alcoholism initiates with episodes of excessive alcohol drinking, known as binge drinking, which is one form of excessive drinking (NIAAA Newsletter, 2004) that is related to impulsivity and anxiety (Ducci et al., 2007; Edenberg et al., 2004) and is also predictive of smoking status. The predisposition of non-alcohol exposed subjects to initiate binge drinking is controlled by neuroimmune signaling that includes an innately activated neuronal Toll-like receptor 4 (TLR4) signal. This signal also regulates cognitive impulsivity, a heritable trait that defines drug abuse initiation. However, the mechanism of signal activation, its function in dopaminergic (TH+) neurons within the reward circuitry implicated in drug-seeking behavior [viz. the ventral tegmental area (VTA)], and its contribution to nicotine co-abuse are still poorly understood. We report that the γ-aminobutyric acid_A_ receptor (GABA_A_R) α2 subunit activates the TLR4 signal in neurons, culminating in the activation (phosphorylation/nuclear translocation) of cyclic AMP response element binding (CREB) but not NF-kB transcription factors and the upregulation of corticotropin-releasing factor (CRF) and tyrosine hydroxylase (TH). The signal is activated through α2/TLR4 interaction, as evidenced by co-immunoprecipitation, and it is present in the VTA from drug-untreated alcohol-preferring P rats. VTA infusion of neurotropic herpes simplex virus (HSV) vectors for α2 (pHSVsiLA2) or TLR4 (pHSVsiTLR4) but not scrambled (pHSVsiNC) siRNA inhibits signal activation and both binge alcohol drinking and nicotine sensitization, suggesting that the α2-activated TLR4 signal contributes to the regulation of both alcohol and nicotine abuse.

## 1. Introduction

Approximately 30% of the current alcohol drinkers in the United States drink alcohol excessively, a condition that kills ~75,000 people annually [1]. Binge drinking (blood-alcohol level ≥ 0.08 g% in a 2-h period) is one form of excessive drinking [2] that is related to impulsivity and anxiety [3,4] and represents a particularly problematic and hazardous form of excessive alcohol intake [5,6]. Interestingly, binge drinking is also predictive of smoking status [7]. Indeed, alcohol shares a high rate of co-abuse with nicotine products that leads to serious health consequences [8]. Furthermore, twin studies suggest that alcohol and nicotine dependence share common genetic mechanisms [9,10,11] that may also contribute to the predisposition to initiate drug abuse. However, the genes that control these two distinct abuse behaviors, particularly as relates to the motivational mechanism that regulates positive reinforcement (binge/intoxication stage) [12], are still unknown. 

Neuroimmune signaling, including the activated innate immunity receptor Toll-like receptor 4 (TLR4), is associated with a lifetime of alcohol consumption [13] and cognitive impulsivity—a heritable trait that correlates with addiction to virtually all drugs of abuse [14,15]. We have previously studied alcohol-preferring (P) rats that display voluntary alcohol consumption [16] and have increased impulsivity, which is believed to represent ethanol-seeking behavior [17,18]. We have shown that the non-alcohol-exposed P rats have an innately activated neuronal TLR4 signal, which is not present in their alcohol-non-preferring (NP) counterparts and is regulated in the central amygdala (CeA) by the α2 subunit of the γ-aminobutyric acid_A_ receptor (GABA_A_R). The activated neuronal TLR4 signal controls the predisposition to initiate binge alcohol drinking, but the signal does not function in the ventral pallidum (VP), documenting the existence of dominant regulatory mechanisms at different brain sites [19,20]. The TLR4 signal is also activated in the ventral tegmental area (VTA), where it upregulates the expression of tyrosine hydroxylase (TH) [21], the rate-limiting enzyme of dopamine (DA) synthesis, which modulates locomotor activity and reward and is an important factor in nicotine addiction [22,23,24]. The innately activated TLR4 signal is sustained by a corticotropin-releasing factor (CRF) amplification loop [20,25] consistent with the CRF function in addiction [12,26,27]. Significantly, the alcohol naïve P rats also readily self-administer more nicotine and express greater nicotine-seeking behavior than the NP rats, and nicotine has greater reinforcing effects in the P rats than the NP rats [28,29], supporting the hypothesis that alcohol and nicotine addiction share common genetic risk factors [11].

The current studies were designed to examine the potential contribution of the activated TLR4 neuronal signal to both alcohol and nicotine abuse. They follow on the findings that (i) alcohol and nicotine activate common neural substrates, including the mesolimbic DA system [30,31,32], (ii) the canonical TLR4 ligand lipopolysaccharide (LPS) does not activate the neuronal TLR4 signal [33] that controls binge drinking, (iii) nicotine alters GABA_A_ receptor signaling pathways in naïve animals [34], and (iv) the majority of DA neurons in the VTA express the GABA_A_R α2 subunit (α2) [35] associated with the activated TLR4 signal [19]. We report that α2 activates the neuronal TLR4 signal, likely through protein–protein interaction, culminating in the activation of the cyclic AMP response element binding (CREB) transcription factor and the upregulation of TH and CRF, both in cultured neuronal cells and in the P rats’ VTA. VTA infusion of α2 or TLR4 siRNA delivered with neurotropic herpes simplex virus (HSV) vectors (amplicons) inhibits alcohol drinking and nicotine sensitization, indicating that the α2-activated TLR4 signal contributes to alcohol and nicotine abuse.

## 2. Materials and Methods

### 2.1. Animals

The alcohol-preferring P rat line fulfills most of the criteria for an animal model of human alcohol abuse to the satisfaction of the alcohol-research community [16,36]. The P rat voluntarily consumes alcohol to attain BALs of 50–200 mg%, lever press (i.e., work) for alcohol orally in concentrations of 10–40%, drink alcohol for its pharmacological effect, develop both tolerance and physical dependence following excessive intake, and upon removal, show signs of withdrawal following chronic consumption [36]. Thus, with specific consideration to the Diagnostic and Statistical Manual of Mental Disorders, fourth edition (DSM-IV) criteria of human alcohol abuse, the P rats represent an ideal model to emulate human binge drinking [16].

These studies are not aimed at examining sex differences and used only male rats; P (*n* = 88; 3–4 months old; 250–550 g) and non-alcohol preferring (NP) rats (*n* = 24, 3–4 months old; 250–550 g) were obtained from the Alcohol Research Center, Indiana University School of Medicine. The P rats perform an operant response for access to ethanol that is not performed by the NP rats, develop both tolerance and physical dependence following excessive intake, and upon removal, show signs of withdrawal following chronic consumption [16]. Animals were individually housed, maintained at an ambient temperature of 21 °C and a reverse 12 h light/dark cycle, and provided with food and water, ad libitum. Training and experimental sessions were conducted between 8:30 a.m. and 5:30 p.m. Treatment was approved by the Institutional Animal Care and Use Committee (IACUC) of the Howard University College of Medicine, and all procedures were conducted in strict adherence with the National Institutes of Health *Guide for the Care and Use of Laboratory Animals*.

### 2.2. Antibodies

The following antibodies were commercially obtained and used according to the manufacturer’s instructions: mouse anti-GAPDH monoclonal [catalog (Cat.) #sc-47724; RRID: AB_627678] and mouse anti-TLR4 monoclonal antibody (Cat. #sc-293072, RRID: AB_10611320) were from Santa Cruz Biotechnology (Santa Cruz, CA, USA). Other used antibodies include rabbit anti-CRF polyclonal (Bioss Antibodies, Woburn, MA, USA; Cat. # bs-0246R, RRID: AB_10885735), mouse anti-TH monoclonal (EMD Millipore, Temecula, CA, USA; Cat. # MAB318, RRID: AB_2201528), rabbit phospho-CREB monoclonal (pCREB; Ser133; Cat. # 9198, RRID: AB_2561044) and rabbit phospho-PKA polyclonal (pPKA; Thr197; Cat. # 4781, RRID: AB_2300165) were from Cell Signaling Technology (Danvers, MA, USA), rabbit anti-NF-kB p65 monoclonal (Abcam, Cambridge, MA, USA, Cat. # ab32536, RRID: AB_776751), mouse beta Actin monoclonal (β-Actin; Proteintech Group, Rosemont, IL, USA, Cat. # 66009-1-Ig, RRID: AB_2687938), Alexa Fluor 488 goat anti-rabbit polyclonal IgG (H+L; Cat. # A11034, RRID: AB_2576217, Thermo Fisher Scientific), and Alexa Fluor 546 goat anti-mouse or goat anti-rabbit polyclonal IgG (H+L; Cat.# A11030, RRID: AB_2534089, or Cat. # A11035, RRID: AB_2534093, respectively, Thermo Fisher Scientific). Horseradish peroxidase-labeled secondary antibodies were anti-rabbit (Cat. # 7074, RRID: AB_2099233, Cell Signaling Technology) and anti-mouse IgG (Cat. # 170-6516, RRID: AB_11125547, Bio-Rad, Hercules, CA, USA). The generation and specificity of the rabbit-derived GABA_A_ α2 polyclonal antibody (W. Sieghart, Center for Brain Research, Medical University of Vienna; Vienna; Austria Cat# GABAA Receptor alpha 2, RRID: AB_2532077) was previously described; it recognizes amino acids 322–357 of the α2 protein [19]. The TLR4, α2, and CRF antibodies are extensively validated [25], and this is further confirmed by the current findings that siRNAs specifically inhibit the expression of their target genes, as determined by immunoblotting, but expression is not inhibited by scrambled siRNA and is restored upon siRNA degradation. 

### 2.3. Cells, Plasmids, Transfection, and Reagents

SK-N-SH (human) and N2a (mouse) neuroblastoma, mouse monocyte/macrophage RAW264.7, and rat pancreatic RINm5F cells were from American Type Culture Collection (Manassas, VA, USA). SK-N-SH and RINm5F cells were grown in RPMI-1640 medium with 2 mM L-glutamine (Gibco, Gaithersburg, MD, USA), 10% fetal bovine serum (FBS; Gemini, West Sacramento, CA, USA), and 1% Penicillin/Streptomycin (Gibco). The N2a and RAW264.7 cells were grown in Dulbecco’s modified Eagle’s medium (DMEM, Gibco) with 10% FBS and 1% Penicillin/Streptomycin. The TLR4^FLAG^ plasmid (# 42646) is a gift from Scott Friedman, and the GABRA2^phGFP^ plasmid (# 49169) is a gift from Tija Jacob and Stephen Moss. The plasmids were from Addgene (Cambridge, MA, USA). They were incubated (15 min, room temperature (RT)) with FuGENE 6 Transfection Reagent (Promega, Madison, WI, USA, Cat. # E2693) in antibiotic-free medium and added to 50–80% confluent cultures (20 µg/T-75 flask, 7 µg/T-25 flask, or 2.6 µg/well of a 6-well plate). The PKA-specific inhibitor H89 (10 μM; Cell Signaling Technology, Cat. # 9844) and the TLR4-specific ligand LPS (1 μg/mL; Sigma-Aldrich, St. Louis, MO, USA, Cat. # L3024) were added to the cultures at 24 h before cell collection.

### 2.4. Immunofluorescence

Immunofluorescent staining was as previously described [20,21,25]. Cells grown on poly-l-lysine (Sigma)-coated glass coverslips (*n* = 5/group) were fixed with 4% paraformaldehyde (PFA) (30 min; RT) and permeabilized (2 min; 4 °C) with 0.1% Triton X-100 in a 0.1% sodium citrate buffer. They were exposed to primary antibodies (diluted in 5% bovine serum albumin and 5% normal goat serum) overnight at 4 °C, washed in phosphate-buffered saline (PBS) with 0.1% Tween 20, and exposed to fluorochrome-labeled secondary antibodies (1 h; RT). For cell surface staining, unfixed (non-permeabilized) cell suspensions were incubated (2 h; 37 °C) with primary antibodies diluted in DMEM without Phenol Red, washed, and exposed (1 h; 37 °C) to Alexa Fluor-labeled secondary antibodies before fixing for image collection. 

For tissue staining, brains were obtained from rats deeply anesthetized by intraperitoneal injection of sodium pentobarbital (Nembutal; 150 mg/kg, Abbott Laboratories, Abbott Park, IL, USA) and transcardially perfused with 0.9% saline followed by 4% PFA in 0.1 M phosphate buffer (PBS, pH 7.4). They were post-fixed in 4% PFA (overnight, 4 °C), incubated in 30% sucrose (48 h, 4 °C), mounted in the optimum cutting temperature (O.C.T.) embedding compound, and frozen at −20 to −40 °C. Free-floating coronal serial sections (30 μm thick) from brain areas containing the whole VTA (extending from −5.0 mm posterior to bregma to −6.0 mm posterior to bregma [37]) were collected from five rats/group using a CM3050 cryostat (Leica, Deerfield, IL, USA) and stored in cryoprotectant (−20 °C) until stained. For each animal, five representative sections from 1:6 series were rinsed in PBS, treated (95 °C, 10 min) with Retrievagen A (BD Biosciences, San Jose, CA, USA, Cat. # 550524), cooled (20 min, RT), and blocked with 5% goat serum (90 min, RT). They were exposed to primary antibodies (overnight, 4 °C) followed by the appropriate Alexa Fluor-labeled secondary antibodies (1 h, RT). For both cultured cells and VTA sections, Z-stack images (1 μm optical steps) were collected on an Olympus Fluoview FV5000 confocal microscope fitted with standard excitation and emission filters [20,21,25]. The total number of single and double staining cells were counted in five randomly selected (×40 magnified) images from each of the five studied sections and the percent of (i) TH+ cells co-expressing TLR4 or α2, (ii) α2+ cells co-expressing TLR4, and (iii) cells with nuclear NF-kB p65 or pCREB staining were calculated for each image. The results are expressed as mean ± SEM.

### 2.5. Immunoblotting

Immunoblotting was as previously described [19,20,21,25,38,39]. Cells grown on T-75 or T-25 flasks (*n* = 5/group) were lysed with radioimmunoprecipitation (RIPA) buffer (20 mm Tris-HCl (pH 7.4), 0.15 mm NaCl, 1% Nonidet P-40 (Sigma, St. Louis, MO, USA), 0.1% SDS (sodium dodecyl sulfate), 0.5% sodium deoxycholate) and supplemented with protease and phosphatase inhibitor cocktails (Sigma). Whole VTA micropunches (300-μm thick) from the naïve P and NP rats and from the P rats infused with pHSVsiTLR4, pHSVsiLA2, or pHSVsiNC amplicons were lysed with CelLytic MT (dialyzable mild detergent, bicine, and 150 mM NaCl; Sigma Aldrich, St. Louis, MO, USA, Cat. # C3228) and supplemented with protease and phosphatase inhibitor cocktails (Sigma) according to the manufacturer’s instructions. The total protein was determined by the bicinchoninic acid assay (BCA, Thermo Fisher Scientific, Waltham, MA, USA, Cat. # 23228 and Cat. # 1859078). The proteins were resolved by SDS–polyacrylamide gel electrophoresis and transferred to polyvinylidene fluoride membranes (PVDF, Bio-Rad, Cat. # 162-0177). Blots were blocked with 5% Blotting-Grade Blocker (Bio-Rad, Cat. # 1706404; for non-phosphorylated primary antibodies) or 5% BSA (for phosphorylated primary antibodies) for 2 hrs at room temperature (RT) and exposed to primary antibody overnight (4 °C), followed by horseradish peroxidase-labeled secondary antibodies for 1 h (RT). Detection was with the Plus-ECL kit reagents (Perkin Elmer, Waltham, MA, USA, Cat. # NEL105001EA) and quantitation was by densitometric scanning with a Bio-Rad GS-700 imaging densitometer.

### 2.6. Co-Immunoprecipitation Assay

N2a cells mock- or α2-transfected were exposed to chemical protein crosslinking [40] at 24 h post-transfection. Briefly, the cells were incubated (20 min on ice) with 1mM of the cleavable, membrane-permeable crosslinker DSP (Thermo Fisher Scientific, Cat. # PG82081). The crosslinker was quenched in 1 M Tris buffer (pH 7.5) (to a final concentration of 10–20 mM), the material was centrifuged at 21,000 g for 15 min, proteins were extracted from the pellet with Pierce IP Lysis Buffer (Thermo Fisher Scientific, Cat. # 87787), supplemented with protease and phosphatase inhibitor cocktails (Sigma), and assayed for co-immunoprecipitation as previously described [38,39]. Specifically, protein lysates (250 µg) were first treated (4 °C; 30 min; on a rocker) with 0.1 µg of normal mouse IgG (EMD Millipore Corporation, San Diego, CA, USA, Cat. # NI03) or normal rabbit IgG (Cell Signaling Technology, Danvers, MA, USA, Cat. # 2729) corresponding to the host species of the primary antibodies together with 20 µL of Protein A/G Plus-Agarose beads (Santa Cruz Biotechnology, Cat. # sc-2003) and Peirce Protein A/G IgG binding buffer (up to 1 mL; Thermo Fisher Scientific, Cat. # 54200). The agarose beads were removed by centrifugation (2500 rpm; 4 °C), and the supernatants were incubated (1 h; 4 °C; on a rocker) with α2 or TLR4 antibodies or normal IgG control (4 μg/each) and Protein A/G Plus-Agarose beads (20 μL) (overnight; 4 °C; on a rocker). The immunoprecipitates were washed four times with ice-cold Pierce IP Lysis Buffer (Thermo Fisher Scientific, Cat. # 87787), and the bound proteins were eluted at 95 °C (5 min) in 50 µL of denaturing solution (150 mM Tris-HCl (pH 7.0), 5.7% SDS, 14% β-mercaptoethanol, 17% sucrose, 0.04% bromthymol blue). Proteins were resolved by SDS-polyacrylamide gel electrophoresis, transferred to PVDF membranes, and immunoblotted with α2 or TLR4 antibodies.

### 2.7. Small Interfering RNAs

Small interfering (si) RNAs were designed to target distinct sequences within the rat TLR4 (Gene bank Entry No: NC_005104.2) or GABA_A_R α2 (Gene bank Entry No: NC_0051 13.2) genes. A scrambled siRNA (siNC) served as a control. The Basic Local Alignment Search Tool (BLAST) search against the EST database (a collection of short single-read transcript sequences from GenBank) was performed to ensure that no other gene was targeted. As previously reported [19,20,21,25], the TLR4 siRNA is AATGCCAGGATGATGCCTC (targets nt −9 to 10), the α2 siRNA is TAAGCTTCCATGAGGACAA (targets nt −9 to 10), and the scrambled siRNA is GCGGCACACGTAGTAAGTT. The siRNAs were synthesized as 60-mer sense and antisense oligonucleotide templates (19 × 2 nt) specific to the targeted genes and 22 nt for restriction enzyme sites and hairpin structure. Synthesis was at the University of Maryland Biopolymer Core Facility and used phosphoramidite (AB) technology. Inhibition of cognate gene expression was confirmed in RAW264.7 cells that express TLR4 and RINm5F cells that express GABA_A_R α2, as previously described [19]. Specifically, the siRNAs were transfected at a final concentration of 65 nM using the siPORT amine transfection agent (Thermo Fisher Scientific, Waltham, MA; Cat# AM4502) according to the manufacturer’s instructions, and extracts collected 72 h post-transfection were immunoblotted with TLR4 or α2 antibodies. 

### 2.8. Herpes Simplex Virus Type 1 (HSV-1)-Based Amplicon Vectors

siRNAs were delivered with non-replicating, non-toxic HSV-1 vectors, known as amplicons. Amplicons are bacterial plasmids that contain two noncoding elements from HSV-1, an origin of DNA replication, and a DNA packaging/cleavage signal, which allow replication and packaging into HSV-1 particles as a 150-kb concatemer. Numerous copies of the transgene sequences are packaged into one vector particle, thereby allowing for high expression levels. Amplicons retain the HSV naturally discriminative in vivo neurotropism, and their use in specific siRNA-mediated gene knockdown was previously described [19,20,21,25]. 

The construction and properties of the amplicons for TLR4 siRNA (pHSVsiTLR4), GABA_A_ α2 siRNA (pHSVsiLA2), and scrambled siRNA (pHSVsiNC) were previously described [19,20,21,25]. Briefly, the pHSVsi vector used to generate the siRNA plasmids that are packaged into HSV-1 virions expresses Enhanced Green Fluorescent Protein (EGFP) under the direction of the HSV-1 IE4/5 immediate-early promoter. The incorporation of EGFP allows for the titration of the vector stocks and the visualization of cell transduction in culture and in the central nervous system (CNS). The pSUPER plasmid, which contains the RNA polymerase III-dependent H1 promoter and well-defined start of transcription and termination signals, is used to generate a second transcription unit for the synthesis of siRNA. The siRNAs were inserted into the pHSVsi vector between the BglII and HindIII sites, downstream of the RNA polymerase III-dependent H1 promoter, and packaged as previously described [19,20,21,25,41]. The amplicon titers were 1 × 10^9^, 5 × 10^8^ and 2 × 10^8^ Transducing Units (TU)/mL for pHSVsiTLR4, pHSVsiLA2, and pHSVsiNC, respectively. 

### 2.9. Stereotaxic Procedures

Amplicon delivery was as previously described [19,20,21]. Briefly, rats were anesthetized through isoflurane/O_2_ gas inhalation and positioned in a stereotaxic apparatus [42]. The microinjection sites in the rat VTA extended from −5.0 mm posterior to bregma to −6.0 mm posterior to bregma, 0.6 mm lateral to the midline in both hemispheres, and −8.2 mm into the brain from the surface of the skull [37]. Because amplicons do not diffuse over long distances, a single large injection would fail to cover the entire site and likely result in a pressure lesion. Accordingly, we gave 9 small injections in each hemisphere spaced across the entire VTA. Each locus received 200 nL of PBS or amplicon (2.5 × 10^5^ TU) delivered with a calibrated pulled-glass micropipette (≈20 μm tip) connected to a Picospritzer II pneumatic pressure injector (Science Products GmbH). Previous studies had shown that similar effects are obtained with 1 × 10^5^ to 1 × 10^6^ TU, and the effects are reversible upon siRNA degradation [19,20,21]. The injections were over 30 s followed by a 1–2-min pause for tissue recovery before insertion of the pipette at the next site. Acrylic microbeads were used to confirm the accuracy of the microinjection placement based on the Rat Brain Atlas [37]. The Institutional Animal Care and Use Committee and Biosafety Committees of Howard University approved the procedures.

### 2.10. Binge Drinking Paradigm

Animals were tested in standard operant chambers (Coulbourn Instruments, Inc., Lehigh Valley, PA, USA) enclosed in an isolated chamber, as previously described [19]. The operant apparatus contained two levers, two dipper manipulanda, triple cue lights over each lever, and a house light. The dipper cup size, which contained the 10% (*v*/*v*) alcohol or 3% (*w*/*v*) sucrose reinforcers, was 0.1 mL. The Coulbourn Graphic State “3” operant software was used.

To emulate human binge drinking, we employed a modification of the drinking-in-the-dark-multiple-scheduled-access (DIDMSA) protocol [19,43]. First, the rats were adapted to a reverse 12 h/12 h light/dark cycle, which began at 7:00 p.m. [lights on] and lasted to 7:00 a.m. [lights off]. They were trained to orally self-administer EtOH daily for two 45-min sessions with 30-min rest in between under a fixed ratio of 1 FR1 schedule employing the sucrose fading technique [42]. After a period of stabilization on the FR1 schedule, the response requirement was then increased to an FR4 schedule, where 4 lever presses are required for access to the reinforcer. For each schedule, responding was considered stable when responses were within ± 20% of the average responses for five consecutive days. Stabilization on the FR4 schedule took approximately eight days. During the stabilization procedures, the animals were never deprived of food or fluid. These procedures are well-established in our laboratory [19,44]. Other cohorts of rats were given a 3% (*w*/*v*) concentration of sucrose and trained in an identical manner under the FR1, then FR4, schedule. Following stabilization on the FR4 schedule for EtOH/sucrose, the DIDMSA protocol began using an FR4 schedule where the rats were given access to 10% alcohol or 3% sucrose on both the left and right levers. To initiate the DIDMSA protocol during the dark phase, the rats were given a 45-min operant session. After the session had elapsed, the rats were then placed in the home cage with food and water ad libitum for 30 min. The rats were then given a second 45-min operant session and subsequently returned to their home cage. The rats engaged in the alcohol drinking for 21 consecutive days.

To ensure that the animals were consuming pharmacologically relevant amounts of EtOH to model human binge drinking [2,6,16], blood alcohol concentration (BAC) was determined in duplicates at 30 and 90 min using previously described procedures [42]. Approximately 100 μL of whole blood was collected from the tail vein into a heparin-coated tube. After collection, the whole blood was immediately centrifuged for 5 min at 1100 rpm. Plasma samples of 5 μL were analyzed in a GL-5 Analyzer (Analox Instruments, Luxenburg, MA, USA). Microanalysis consisted of measuring the oxygen consumption in the reaction between the sample of alcohol and alcohol oxidase using a Clark-type amperometric oxygen electrode. Alcohol reagent buffer solutions (pH 7.4) and alcohol oxidase enzymes were used in all samples tested. This BAC measurement is consistent with the National Institute on Alcohol Abuse and Alcoholism (National Institutes of Health) (NIAAA) definition of binge alcohol consumption in humans [16,45]. Using this protocol, the rats produced consistent BACs of 99 ± 3 mg% after 90 min of drinking. Sucrose control rats were trained in a similar manner, but lever pressed for a 3% sucrose solution instead of ethanol. The sucrose control rats allowed for evaluation of reinforcer specificity.

### 2.11. Kelley Nicotine Sensitization Paradigm

The Kelley model is well-established in the nicotine sensitization literature [46,47]. The P and NP rats were habituated to PBS subcutaneous (sc) injections and locomotor activity sessions for five consecutive days (Phase I). They were then subjected to nicotine sc injections (0.4 mg/kg) for seven consecutive days and locomotor activity (horizontal activity (ambulation); 90 min total session time) was measured (Phase II). In the P rats treated with amplicon vectors, the vectors were infused into the VTA, and after 72 h of recovery, the rats were subjected to nicotine injections (Phase III) for 10–15 days and locomotor activity was measured. 

### 2.12. Statistics

Data were analyzed by two-way ANOVAs for alcohol/sucrose drinking and nicotine sensitization or paired *t*-tests for molecular assays, respectively performed using the SigmaPlot 11.2 and Prism 5.0c software programs. Significant ANOVAs were followed by Newman–Keuls post-hoc tests for the Kelley nicotine sensitization assay, where there was a significant interaction of Line × Day. *p* < 0.05 is considered statistically significant.

## 3. Results

### 3.1. The P Rats Have Larger Numbers of VTA-Located TH+ Neurons that Co-Express TLR4 and α2 Than the NP Rats

**Figure 1:**
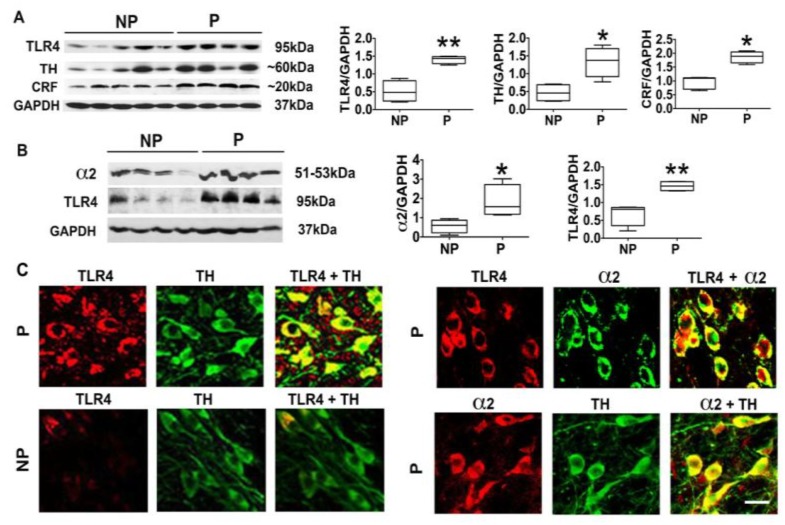
The alcohol-preferring (P) rats have a higher percentage of ventral tegmental area (VTA) TH+ neurons that co-express Toll-like receptor 4 (TLR4) and the γ-aminobutyric acid_A_ receptor (GABA_A_R) α2 subunit (α2) than alcohol-non-preferring (NP) rats. (**A**) VTA micropunches were collected from the naïve (not drug-exposed) NP (*n* = 5) and P (*n* = 4) rats, and protein extracts were immunoblotted with antibodies to TLR4, tyrosine hydroxylase (TH), corticotropin-releasing factor (CRF), and GAPDH used as gel loading control. The results are expressed as densitometric units normalized to GAPDH ± SEM, as described in Section 2, and each lane represents an animal. The TLR4, TH, and CRF levels are higher in the P rats than the NP rats. (* *p* ≤ 0.05; ** *p* ≤ 0.01 by *t*-test). (**B**) Protein extracts duplicate VTA micropunches from the NP (*n* = 4) and P (*n* = 4) rats were immunoblotted with α2, TLR4, and GAPDH antibodies, and the results were quantitated as described above. The α2 and TLR4 levels are higher in the P rats than the NP rats. (* *p* ≤ 0.05; ** *p* ≤ 0.01 by *t*-test). (**C**) VTA sections from the P and the NP rats (*n* = 5/group) were stained in double immunofluorescence with antibodies to (i) TLR4+TH, (ii) TLR4+α2, or (iii) TH+α2, examined by confocal microscopy and Z-stack imaging. Scale bars are 20 μm.

We have previously shown that the P rats have an α2-regulated TLR4 signal in the CeA that controls the predisposition to initiate alcohol drinking [19]. However, the relationship of α2 to the TLR4 signal at brain sites involved in the reward circuitry that affects all addictive behaviors, such as the VTA [12,48], is still unknown. Two series of experiments were done in order to begin to address this question. First, VTA punch biopsies were collected from P (*n* = 8) and NP (*n* = 9) rats that had not been previously exposed to alcohol, and protein extracts were immunoblotted with antibodies to TLR4, stripped, and sequentially re-probed with antibodies to α2, TH, CRF, and GAPDH, used as gel loading control. The results were quantitated by densitometric scanning and are expressed as densitometric units normalized to GAPDH, as previously described [19,20,21,25,49]. As shown in Figure 1, the P rats have significantly higher levels of TLR4 at this site than the NP rats (*p* ≤ 0.01), and this is accompanied by significantly (*p* ≤ 0.05), increased levels of α2 (Figure 1B), TH (Figure 1A), and CRF (Figure 1A).

Because (i) TLR4 is known to contribute to the addiction-related reward system activity [50], (ii) TH is the rate-limiting enzyme for DA synthesis [51], and (iii) we have previously shown that CRF is a target of the activated TLR4 signal that feedback controls TLR4 expression [25], the second series of experiments asked whether the percentage of dopaminergic (TH+) neurons that co-express α2 and TLR4 is significantly higher in the P rats than in the NP rats. VTA sections from the P and NP rats were stained in double immunofluorescence with antibodies to TLR4 and TH, and duplicate sections from the P rats were stained with antibodies to α2 + TLR4 or α2 + TH. The percent of staining cells was calculated as described in Section 2. As previously reported by Okada et al. (2004) [35], α2 was expressed by most (85 ± 3.1%) of the TH+ cells. TLR4 staining was seen in 61 ± 5.2% of the TH+ neurons from the P rats as compared to 11 ± 5.2% of the TH+ cells in the NP rats (*p* < 0.05), and 60 ± 6.3% of the α2+ cells in the P rats co-expressed TLR4 (Figure 1C). Collectively, the data indicate that TLR4 co-localizes with α2 in a significantly higher percentage of TH+ neurons in the VTA from the P rats than that from the NP rats, and this is consistent with the higher levels of protein expression in the P rats. 

### 3.2. The Neuronal TLR4 Signal is Activated by α2 and it Culminates in Activated CREB, but not NF-kB

**Figure 2:**
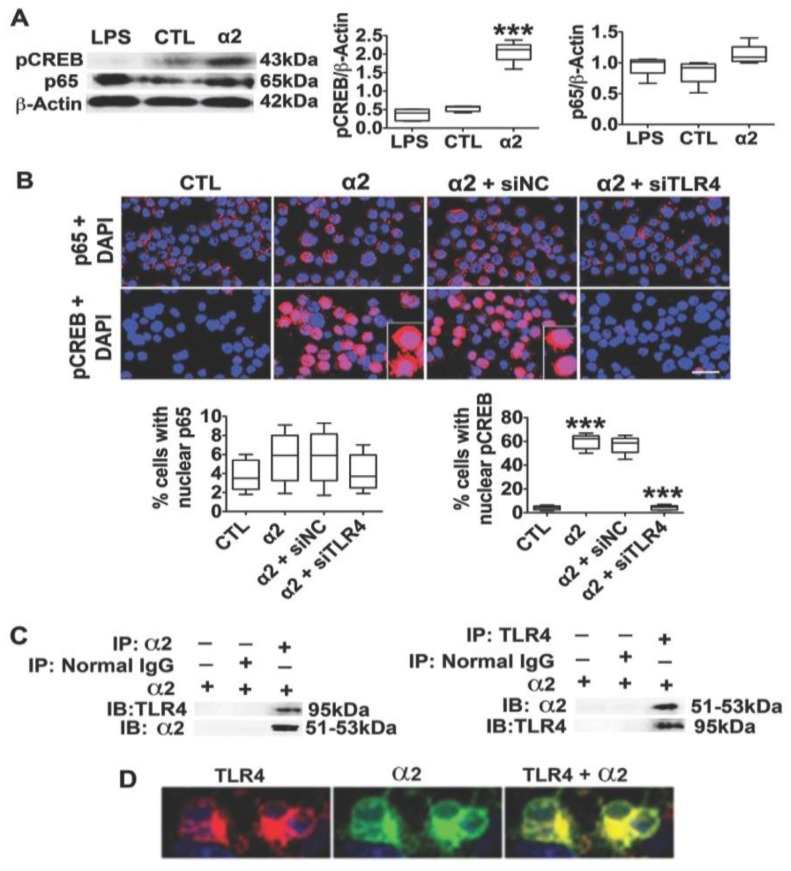
α2 binds TLR4 and activates its signal in neurons. (**A**) Protein extracts from N2a cells untreated (CTL) and cells treated with LPS (1 µg/mL) or transfected with α2 (24 h post-treatment) were immunoblotted with antibodies to pCREB NF-kB p65 and β-Actin, used as gel loading control. The results were quantitated by densitometric scanning and expressed as densitometric units normalized to β-Actin. pCREB levels were significantly (*** *p* ≤ 0.001 by *t*-test) increased by α2, but not LPS. NF-kB p65 expression was not altered by either treatment. (**B**) Untransfected N2a cells (CTL) or cells transfected with α2 (24 h) in the presence or absence of pHSVsiTLR4 or pHSVsiNC amplicons were stained with pCREB or NF-kB p65 antibodies (red) and examined for nuclear localization (activation). DAPI (blue) was used as nuclear counterstain. α2 transfection significantly increased the percentage of cells with nuclear pCREB but not NF-kB p65 staining, and pCREB nuclear staining was inhibited by pHSVsiTLR4 (siTLR4) but not the pHSVsiNC (siNC) (*** *p* ≤ 0.001 by *t*-test). Scale bar is 30 μm. (**C**) Protein extracts from N2a cells transfected with α2 were immunoprecipitated (IP) with the α2 or TLR4 antibodies or normal IgG (control), and the precipitates were reciprocally immunoblotted (IB) with α2 or TLR4 antibodies. Both α2 and TLR4 were seen in the anti-α2 and anti-TLR4 (but not normal IgG) precipitates from the transfected cells, indicative of protein–protein interaction. (**D**) Immunofluorescent staining of non-permeabilized α2-transfected N2a cells with TLR4 (red) and α2 (green) antibodies indicates cell surface co-localization (yellow).

Previous studies have shown that the canonical TLR4 ligand LPS does not activate TLR4 signaling pathways in neurons, but the mechanism of signal activation in these cells is unknown [33]. Having seen that α2 co-localizes with TLR4 in a high percentage of TH+ neurons from the P rats but not the NP rats, we wanted to know whether α2 is involved in TLR4 signal activation in neurons. N2a cells that express TLR4 were treated with LPS (1 μg/mL) or transfected with the α2 plasmid, and protein extracts collected at 24 h post-treatment were immunoblotted with antibodies to the TLR4-activated transcription factors NF-kB p65 and pCREB [33,52] followed by β-Actin, used as gel loading control. The results were quantitated by densitometric scanning and are expressed as mean, β-Actin-adjusted, densitometric units ± SEM. The levels of pCREB and NF-kB p65 were similar in the LPS- and mock-treated N2a cells, but the α2-transfected cells had significantly (*p* ≤ 0.001) higher levels of pCREB than those in the mock-transfected cells (Figure 2A). The increased pCREB levels reflect signal activation, because (i) the percent of cells with pCREB nuclear translocation also showed a significantly greater increase for the α2-transfection than for the mock-transfection (62.2 ± 3.6% and 4.3 ± 2.1%, respectively; (*p* ≤ 0.001)) and (ii) nuclear translocation was inhibited by the TLR4-specific (siTLR4) but not scrambled (siNC) siRNA amplicons (Figure 2B). We conclude that pCREB is a specific target of the α2-activated TLR4 signal, because the NF-kB p65 levels were similar in the α2- and mock-transfected cells (Figure 2A), and it did not translocate to the nucleus (Figure 2B). Collectively, the data indicate that the neuronal TLR4 signal is activated by α2, resulting in the specific activation of CREB, but not NF-kB. 

### 3.3. α2+ Binds TLR4, Likely at the Cell Surface

Signaling pathways and biological function are regulated by protein–protein interaction, often involving specific protein domains [53,54,55]. To examine whether α2 activates the TLR4 signal through its ability to bind TLR4, N2a cells were transfected (or not) with the α2 plasmid and protein extracts (24 h post-transfection) were studied for TLR4/α2 co-precipitation as previously described [38,39] and detailed in Section 2. The protein extracts were immunoprecipitated with α2 or TLR4 antibodies followed by reciprocal immunoblotting with both antibodies. Immunoprecipitation with normal IgG served as the control. The data summarized in Figure 2C indicate that α2 and TLR4 were present in the reciprocal TLR4 or α2 precipitates but not in the normal IgG precipitates, indicating that α2 binds TLR4. Binding likely occurs at the cell surface, because the immunofluorescent staining of non-permeabilized (unfixed) cells indicated that the cells stain with both the TLR4 and α2 antibodies and the staining was co-localized (Figure 2D). Collectively, the data indicate that α2 binds TLR4, likely contributing to signal activation. However, the potential contribution of proteins that serve as ligands or scaffolds to facilitate binding is still unknown.

### 3.4. The α2-Activated TLR4 Signal Contributes to CRF and TH Expression

**Figure 3:**
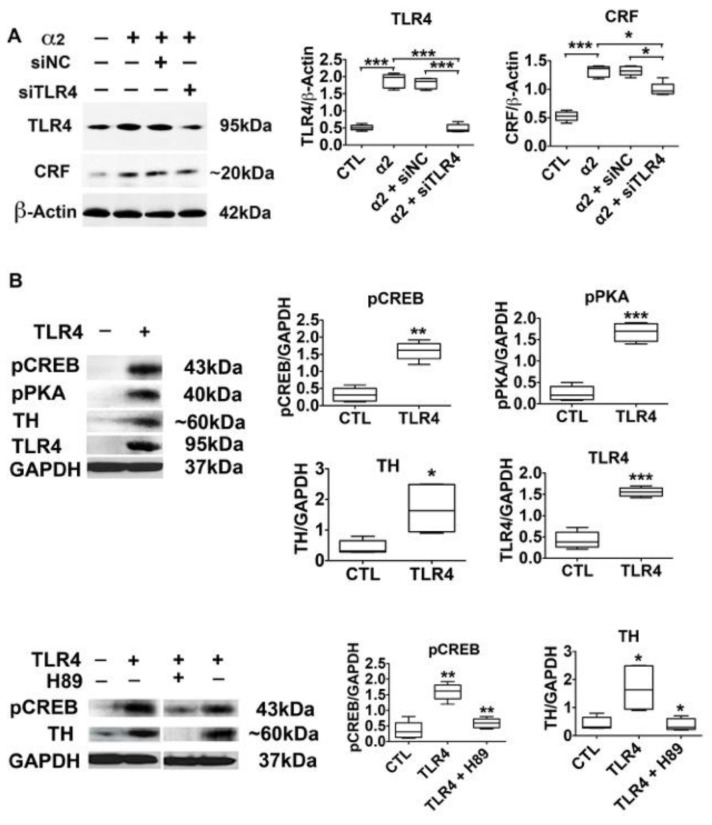
The α2-activated TLR4 signal controls CRF and TH expression (**A**) Protein extracts from N2a cells mock- or α2-transfected in the presence or absence of pHSVsiTLR4 or pHSVsiNC (*n* = 5/group) were immunoblotted with antibodies to CRF, TLR4, or β-Actin, and the results are expressed as densitometric units normalized to β-Actin ± SEM. The levels of CRF and TLR4 are significantly higher in the α2- than in the mock-transfected cells, and upregulation is inhibited by siTLR4 but not siNC (* *p* ≤ 0.05, *** *p* ≤ 0.001 by *t*-test). (**B**) Protein extracts from mock- or TLR4-transfected SK-N-SH cells (*n* = 5 each) were immunoblotted with antibodies to pCREB, pPKA, TH, TLR4, or GAPDH, and the results are expressed as GAPDH-normalized densitometric units ± SEM. The levels of pCREB, pPKA and TH are significantly higher in the TLR4- than in the mock-transfected cells. The pCREB and TH upregulation is inhibited by treatment with the PKA-specific inhibitor H89 (10 μM) (* *p* < 0.05; ** *p* < 0.01; *** *p* < 0.001 by *t*-test).

Having seen that the α2 activated TLR4 signal culminates in CREB activation, we wanted to know whether it targets genes with promoters that contain cyclic AMP response elements (CRE) that are involved in binge drinking and nicotine sensitization. Two series of experiments were done. First, we focused on CRF, which has CRE promoter motifs [56,57,58] and sustains the activated neuronal TLR4 signal through CRFR1 and the MAPK/ERK pathway [25]. N2a cells were transfected with the α2 plasmid in the presence or absence of the TLR4 (pHSVsiTLR4) or scrambled (pHSVsiNC) siRNA amplicons, and protein extracts collected at 24 h post-treatment were immunoblotted with antibodies to TLR4 or CRF followed by β-Actin, used as gel loading control. The results were quantitated by densitometric scanning and are expressed as mean β-Actin-adjusted densitometric units ± SEM. α2 transfection significantly increased the levels of CRF and TLR4, an increase that was inhibited by TLR4 but not scrambled siRNA (Figure 3A). The data indicate that the α2- controlled TLR4 signal is involved in regulating CRF expression, but the potential contribution of other factors cannot be excluded.

In the second series of experiments to examine the contribution of α2 to the activation of the TLR4 signal, we focused on TH, the expression of which characterizes the VTA dopaminergic neurons that express TLR4 (Figure 1). The TH promoter contains canonical and non-canonical CRE motifs that bind CREB and modulate expression [59,60,61], and it is the rate-limiting enzyme for DA synthesis, an important factor in nicotine addiction [22,23,24]. Because the activated (phosphorylated) PKA (pPKA, Thr197) in turn activates CREB by phosphorylation at Ser-133 (pCREB) [62,63,64], the effect of the PKA-specific inhibitor H89, was studied in parallel. SK-N-SH cells, which are essentially TLR4 negative, were transfected with a TLR4 plasmid in the presence of the PKA-specific inhibitor H89 (10 μM). Protein extracts collected 24 h post-treatment were immunoblotted with TH antibody, and the stripped blots were re-probed with antibody to TLR4 to confirm transfection. This was followed by immunoblotting with antibodies to pPKA, its target pCREB, and GAPDH, used as gel-loading control. The results were quantitated by densitometric scanning, and are expressed as mean GAPDH-adjusted densitometric units ± SEM. The levels of TH were significantly (*p* ≤ 0.05) higher in the TLR4- than in the mock-transfected cells, and this was accompanied by a significant (*p* ≤ 0.01) increase in the levels of pCREB (Figure 3B). The PKA-specific inhibitor H89 inhibited the upregulation of both pCREB and TH (Figure 3B), indicating that the activated TLR4 signal targets TH through PKA/CREB activation. 

**Figure 4:**
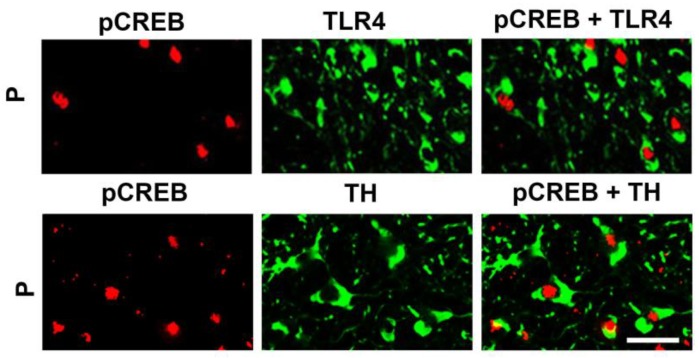
TH+ neurons in the VTA co-express TLR4 and nuclear pCREB. Confocal microscopy and Z-stack imaging of double immunofluorescent staining for pCREB + TLR4 or pCREB + TH is shown for the VTA of the P rats (*n* = 5/group). Merged images for pCREB (red) and TH (green) reveal TH+ neurons that co-express intranuclear pCREB, and merged images for pCREB and TLR4 reveal TLR4+ neurons that co-express intranuclear pCREB. Scale bars are 20 μm.

Significantly, CREB is also activated in the VTA from the P rats, as evidenced by pCREB nuclear translocation in a relatively high proportion of TLR4+ and TH+ cells, as determined by double immunofluorescent staining with pCREB and TLR4 or TH antibodies (51.9 ± 5.8% and 54.5 ± 5.6% of the cells, respectively) (Figure 4). Consistent with our previous findings for wild-type rats [21], nuclear staining with pCREB antibody was not seen in the VTA from the NP rats (data not shown). Collectively, the data indicate that the α2-activated TLR4 signal targets CRF and TH expression through CREB signaling.

### 3.5. The α2/TLR4 Signal Controls Binge Drinking

**Figure 5:**
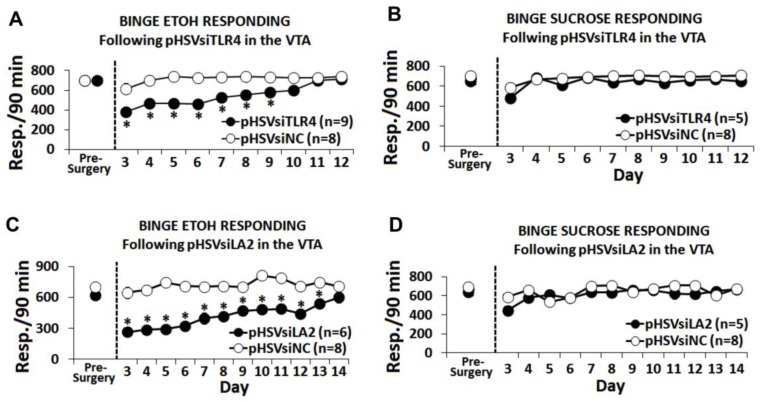
The α2/TLR4 signal in the VTA regulates binge alcohol drinking. pHSVsiTLR4 (**A**) and pHSVsiLA2 (**C**) microinjection in the VTA from the P rats reduced binge alcohol drinking on days 3–11 and 3–14 post-surgery, respectively. pHSVsiTLR4- or pHSVsiLA2-infused rats had virtually no alcohol intake on days 3–6 after surgery. Thereafter, drinking increased with time, returning to the original pre-surgery levels on days 12–14 after infusion, respectively. pHSVsiNC did not alter responding for alcohol (**A,C**) (* *p* ≤ 0.05). Microinjection of pHSVsiTLR4 and pHSVsiNC (**B**) or pHSVsiLA2 and pHSVsiNC (**D**) into the VTA did not alter responding for sucrose. Average responding for (**A**) 10% alcohol and (**B**) 3% sucrose after a 90-min operant session prior to and for 10 days following the infusion of pHSVsiTLR4 or pHSVsiNC (scrambled amplicon) into the VTA. Significant Treatment x Day effects were seen following pHSVsiTLR4 treatment for alcohol drinking (*F*(20,80) = 3.957, *p* < 0.001) but not for sucrose drinking (*F*(10,40) = 0.761, *p* = 0.664). Significant Treatment x Day effects were also seen following pHSVsiLA2 treatment for alcohol drinking (*F*(12,60) = 4.839, *p* <0.001) (**C**) but not for sucrose drinking (*F*(12,48) = 1.288, *p* = 0.257) (**D**).

To examine the role of the VTA α2/TLR4 signal in binge alcohol drinking, we used the operant DIDMSA protocol developed by the Integrative Neuroscience Initiative on Alcoholism (INIA-West) [16,36]. Using this protocol, blood-alcohol levels were ≥110 mg/dL. The rats trained to binge on sucrose (0.1% wt/vol) on a similar schedule were studied in parallel. Cohorts of the P rats trained to self-administer alcohol were randomly given, by cohort, pHSVsiLA2, pHSVsiTLR4, or pHSVsiNC into the VTA by bilateral stereotaxic infusion. After three days, during which the animals were allowed to recover from the stress of surgery, they were given the opportunity to engage in alcohol (Figure 5A,C) or sucrose (Figure 5B,D) drinking and were examined daily for 12–14 days. On days 3–6 after surgery, alcohol intake was dramatically reduced by both pHSVsiTLR4 (Figure 5A) and pHSVsiLA2 (Figure 5C). Thereafter, drinking increased with time, returning to the original pre-surgery levels on days 12–14 after infusion. This finding is in direct contrast to rats given pHSVsiNC, who evidenced minimally reduced alcohol drinking only during the first three days after surgery. Throughout the next 12–14 days of follow-up, the pHSVsiNC-treated rats displayed baseline (pre-surgery) levels of alcohol drinking (Figure 5A,C). Sucrose responding was not affected by pHSVsiTLR4 or pHSVsiLA2 microinjection (*p* > 0.05; Figure 5B,D), confirming the specificity of their activity for alcohol drinking. 

**Figure 6:**
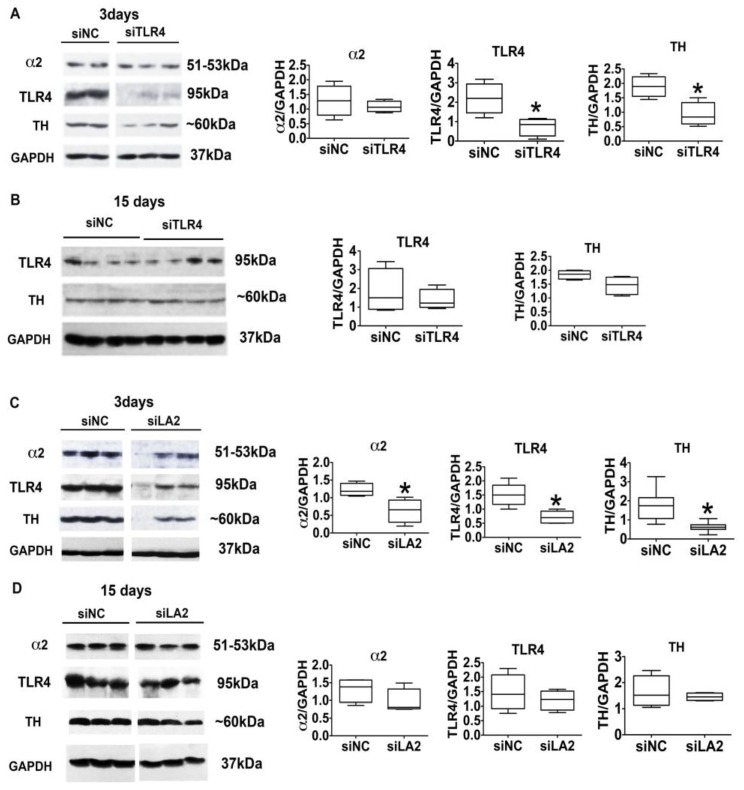
pHSVsiTLR4 and pHSVsiLA2 inhibit TLR4, α2, and TH expression in the VTA. pHSVsiTLR4 (**A**) and pHSVsiLA2 (**C**) but not pHSVsiNC (**A**,**C**) inhibit TLR4 and TH expression in the VTA on day 3 post-treatment. pHSVsiLA2 (**C**) inhibits α2, TLR4 and TH expression on day 3 post infusion (* *p* ≤ 0.05 by *t*-test). The expression of TLR4, α2, and TH is restored to the levels seen in pHSVsiNC-treated animals (innate levels) on day 15 after pHSVsiTLR4 (**B**) or pHSVsiLA2 (**D**) infusion (*p* > 0.05 by *t*-test).

We conclude that binge drinking is controlled by the α2-activated TLR4 signal, because the signal targets TH, the expression of which was also controlled by the pHSVsiLA2 and pHSVsiTLR4 amplicons. Indeed, immunoblotting of protein extracts from VTA tissues collected at three days post-treatment with pHSVsiTLR4 or pHSVsiLA2, when drinking was maximally decreased, indicated that the levels of TLR4 and TH were significantly (*p* ≤ 0.05) lower than those seen in animals similarly treated with pHSVsiNC, which did not reduce drinking (Figure 6A,C). Consistent with the conclusion that α2 is upstream of TLR4, pHSVsiLA2 reduced the expression of α2, TLR4, and TH (Figure 6C), but pHSVsiTLR4 only reduced the expression of TLR4 and TH but not α2 (Figure 6A). On day 15 post-treatment, when alcohol drinking was no longer inhibited, the levels of α2, TLR4, and TH in the pHSVsiLA2- and pHSVsiTLR4-treated animals were restored to those seen in the ones given pHSVsiNC, confirming the regulatory activity of the α2-activated signal (and the specificity of the studied antibodies) (Figure 6B,D). The duration of the amplicon inhibitory effect is consistent with that previously reported and likely reflects the duration of siRNA integrity/ availability (10–14 days) and the resulting posttranscriptional gene silencing [19,41]. Collectively, the data indicate that the α2/TLR4 signal in the VTA controls binge drinking, which is one form of excessive drinking [2] that is related to impulsivity and anxiety [3,4]. Notably, however, α2/TLR4 binding and the resulting TLR4 signal activation occur in non-alcohol exposed cultured cells and rats, indicative of its function independent of alcohol and preceding exposure to its effects, suggesting that it may contribute to the predisposition to engage in drinking. 

### 3.6. The α2/TLR4 Signal in the VTA Controls Nicotine Sensitization

**Figure 7:**
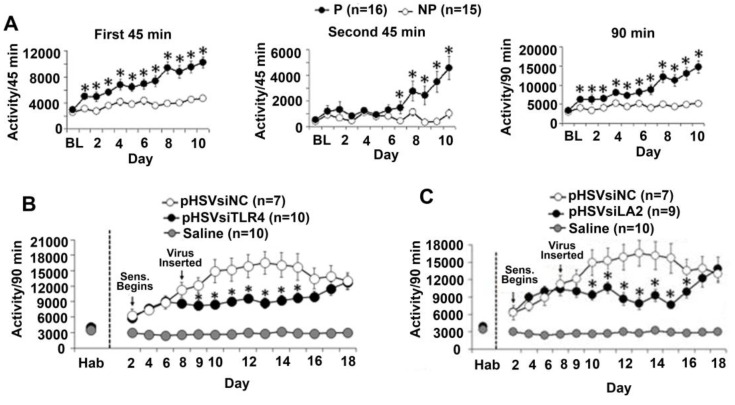
Nicotine enhances locomotor sensitization in the P rats through α2/TLR4. (**A**) Subcutaneous (sc) injections of nicotine (0.4 mg/kg) immediately before the daily locomotor activity examination showed that nicotine sensitization occurs in the P (*n* = 16) rats but not in the NP (*n* = 15) rats. Compared with the NP rats, the P rats were sensitive to the locomotor-enhancing effects of nicotine, with a significant interaction of Line × Day during the first 45-min (*F*(11,154) = 8.723, *p* < 0.001), second 45-min (*F*(11,154) = 8.805, *p* < 0.001), and summative 90-min periods (*F*(11,154) = 4.047, *p* < 0.001). (**B**,**C**) The P rats habituated to saline were infused with pHSVsiTLR4 (*n* = 10) (**B**), pHSVsiLA2 (*n* = 9) (**C**), or pHSVsiNC (*n* = 7) (**B**,**C**) used as a control in the VTA, and after 72 h of recovery, they were given sc injections of nicotine (0.4 mg/kg) and examined for locomotor activity. Saline (*n* = 10) was infused alone, and locomotor activity was measured in parallel, with saline infusion showing a baseline of locomotor activity (**B**,**C**). Horizontal activity of the P rats during nicotine sensitization was significantly decreased by treatment with pHSVsiTLR4 (**B**) or pHSVsiLA2 (**C**) but not pHSVsiNC (**B**,**C**). Significant Treatment × Day effects were seen following both pHSVsiTLR4 (*F*(13, 94) = 3.543, *p* < 0.001) and pHSVsiLA2 (*F*(13,89) = 4.232, *p* < 0.001) treatments.

Nicotine-induced behavioral sensitization is a potential measure of vulnerability to nicotine dependence that complements nicotine self-administration [65]. To examine whether the α2/TLR4 signal also regulates nicotine sensitization, we measured locomotor activity using an established protocol that focuses on horizontal movement, which is considered to be the most reliable of all activity measures [46,47]. The P and NP rats were habituated to saline, given sc injections of nicotine (0.4 mg/kg), and examined for locomotor activity as described in Section 2. Compared to the NP rats, the P rats were sensitive to the locomotor-enhancing effects of nicotine, with a significant interaction of Line × Day during the first 45-min (*F*(11,154) = 8.723, *p* < 0.001), second 45-min (*F*(11,154) = 8.805, *p* < 0.001), and summative 90-min periods (*F*(11,154) = 4.047, *p* < 0.001) (Figure 7A). To examine the contribution of the α2/TLR4 signal, saline-habituated P rats were infused with the pHSVsiTLR4, pHSVsiLA2, or pHSVsiNC amplicons in the VTA, given sc injections of nicotine (0.4 mg/kg) after 72 h of recovery, and examined for locomotor activity. Horizontal activity of the P rats during nicotine sensitization was significantly decreased by pHSVsiTLR4 (Figure 7B) or pHSVsiLA2 (Figure 7C) but not pHSVsiNC infusion into the VTA. Significant Treatment × Day effects were seen following both pHSVsiTLR4 (*F*(13, 94) = 3.543, *p* < 0.001) and pHSVsiLA2 (*F*(13,89) = 4.232, *p* < 0.001) treatments, but the effect was no longer seen on day 15 post-treatment. Together with the finding that the pHSVsiLA2 and pHSVsiTLR4 amplicons inhibit the α2-activated TLR4 signal in cultured cells, as well as in the VTA, the data indicate that the signal also regulates nicotine vulnerability in the P rats. 

## 4. Discussion

The salient feature of the data presented in this report is the finding that α2 is a specific activator of TLR4 signaling in neurons, that culminates in the activation of CREB but not NF-kB and results in the upregulation of CRF and TH expression. The signal is likely activated through α2/TLR4 protein interaction, and it regulates binge alcohol drinking and nicotine sensitization. The following comments seem pertinent with respect to these findings. 

The combined use of alcohol and tobacco is a common behavior. The average number of consumed cigarettes is significantly higher among alcoholic than nonalcoholic smokers, alcohol-dependent individuals have higher rates of nicotine dependence than non-alcohol drinking subjects, and alcohol intake is significantly higher in users that are also smokers [8]. Common genetic mechanisms are believed to contribute to drug dependence [9,10,11], and genes in several neurotransmitter pathways were suggested as potential candidates [66,67]. However, the identity of the genes that predispose to drug co-abuse and their function at brain sites involved in the reward circuitry are still poorly understood. 

Neuroimmune signaling has been implicated in excessive alcohol intake [13]. Intraperitoneal injection of the TLR4-canonical ligand, LPS, increases the duration of ethanol self-administration [68], an effect mediated by inflammatory pathways [69], and drug-mediated blocking of TLR4 activation reduces alcohol drinking and alcohol-induced neuroimmune responses in alcohol-dependent mice [70]. TLR4 knockout (KO) mice have reduced alcohol preference following intermittent intraperitoneal treatment [71], and both TLR4 and TLR2 were implicated in excessive alcohol drinking in KO mice [72]. However, information generated through the use of KO animals is potentially misleading, as the organism is known to upregulate one or more genes that modulate the affected knocked-out function [73,74]. This is certainly the case for TLRs, which share many properties and functions [75]. Nonetheless, a recent study designed to address the role of neuroimmune signaling in alcohol drinking concluded that TLR4 has minimal impact on the amount of consumed alcohol, but it mediates the acute sedative effects, which might indicate the susceptibility to develop alcohol dependence [76]. 

Our studies were not designed to examine the effect of the TLR4 signal and its specific genes on the amount of consumed alcohol but rather their effect on the susceptibility to engage in drug abuse, specifically focusing on binge alcohol drinking and nicotine sensitization. They used siRNA-mediated gene knockdown and were done in alcohol-preferring P rats that satisfy the essential criteria for an animal model of addictive behavior. Gene knockdown with siRNA has the advantage of specifically targeting a gene of interest at a specific site within an otherwise unaltered genetic makeup and of lacking the compensatory response seen in KO mutants [73]. Moreover, the siRNA inhibitory effect is temporary, defined by the duration of siRNA integrity/availability [19,41], providing a built-in control for the actual contribution of the targeted gene. Relative to their NP counterparts, the P rats display voluntary alcohol consumption [16,36], and they have increased impulsivity, a heritable trait that represents the alcohol seeking behavior [17,18]. They also readily self-administer more nicotine and express greater nicotine-seeking behavior than the NP rats, and nicotine has greater reinforcing effects in the P rats than the NP rats [29]. Importantly, given their development by selective breeding rather than genetic manipulation (viz. KO or mutation), the P rats do not have the KO/mutant-associated genetic alterations that preclude a better understanding of the naïve non-exposed individual predisposed to initiate drug abuse. 

Using siRNA-mediated gene knockdown, we have previously shown that the P rats have an innately activated TLR4 signal that is located in neurons and is not present in the NP rats. This neuronal TLR4 signal is innately activated in the P rats, and it regulates the predisposition to initiate binge alcohol drinking and cognitive impulsivity. It is sustained, in the absence of alcohol, by a CRF/CRFR1 amplification loop, and in the CeA, it is downstream of the GABA_A_R α2 subunit [19,20,21,25]. However, the mechanism whereby α2 regulates the activation of this neuronal TLR4 signal, its function in the VTA, and its potential role in alcohol/nicotine abuse are still unclear. This is particularly relevant, because alcohol and smoking influence the expression of LPS-activated pro-inflammatory cytokines in non-neuronal cells/tissues [77,78], but LPS does not activate the neuronal TLR4 signal [33] that regulates impulsivity and the predisposition to alcohol binge drinking [21,25].

Consistent with previous findings that GABA transmission modulates DA neuronal activity [79], we show that TLR4 is co-expressed with α2 in dopaminergic (TH+) neurons, the percentage of which is significantly higher in the P rats than in the NP rats. Moreover, α2, but not LPS, activates the TLR4 signal in neuronal cells, and it specifically results in the activation of CREB but not NF-kB. CREB activation is through PKA, as confirmed both by phosphorylation (increased pPKA and pCREB levels) and pCREB nuclear translocation, and it regulates the expression of CRF and TH, both of which are pCREB targets. Signal activation is inhibited by TLR4 and α2 siRNA but not scrambled siRNA, and both PKA and CREB phosphorylation is inhibited by the PKA-specific inhibitor H89. This is not an in vitro artefact, because (i) the levels of TLR4, α2, TH, and CRF are also significantly higher in the VTA from the P rats than from the NP rats, (ii) α2 co-localizes with TLR4 in a high percentage of TH+ neurons at this site, (iii) TLR4+ neurons in the VTA from the P rats evidence nuclear pCREB localization, and (iv) VTA infusion of the pHSVsiTLR4 or pHSVsiLA2, but not pHSVsiNC, amplicons reduces signal activation in the P rats. The use of amplicons to deliver the siRNAs insures neuronal targeting, because amplicons retain the HSV naturally discriminative neurotropism after CNS administration. Indeed, HSV establishes life-long infection of the peripheral ganglia sensory neurons, known as latency, and it causes encephalitis associated with neuronal apoptosis [19,20,80,81,82,83,84,85,86,87,88,89]. 

The finding that the α2-regulated TLR4 signal specifically activates (phosphorylates) PKA/CREB and upregulates CRF and TH expression supports the interpretation that it is likely to regulate alcohol and nicotine abuse. Indeed, pPKA/pCREB was implicated in alcohol drinking [90], and acute nicotine treatment was shown to increase the levels of pCREB in the VTA [91]. Stress, which is involved in addiction and impulsivity, upregulates CRF, which plays a key role in excessive, dependence-like alcohol drinking [12,26,27,49,92]. CRF signaling through CRFR1 is involved in cross-sensitization to psychostimulants [93], and the CRF/CRFR1 system sustains the innately activated TLR4 signal in the NAc shell from the naïve P rats that regulates impulsivity [25]. TH is the rate-limiting enzyme of DA synthesis and its increased expression is an important factor in nicotine addiction [22,23,24]. Our recent studies using protein extracts from the P rats’ VTA micoropunches, confirm that α2 binds TLR4 at this site, as determined by co-immunoprecipitation, and binding is associated with CREB activation (Balan et al. submitted [94]). Moreover, the finding that α2/TLR4 are co-expressed in dopaminergic (TH+) neurons is consistent with previous reports that (i) the activated TLR4 signal is located in TH+ neurons, the firing rate of which in the VTA is increased by acute nicotine administration, and (ii) increased DA transmission is involved in the reinforcing effect of nicotine [21,31,95,96]. 

How does α2 activate the TLR4 signal? The answer to this question is still unclear. Protein–protein interaction is known to regulate response. Dopamine D1 and CRFR2a assemble into functionally interacting complexes [97], and receptor–receptor interactions can regulate response diversity and bias [98]. TLR4 is known to bind multiple proteins, and it has been suggested that the resulting combinatorial signaling may account for a broad response repertoire [99,100]. Alcohol induces TLR4/TLR2 heterodimer formation in microglia [101], and TLR4/TLR6 binding was shown to initiate inflammatory signaling events [102]. Our co-immunoprecipitation studies using fully validated antibodies indicate that α2 binds TLR4 in neuronal cells, and immunofluorescent staining suggests that binding occurs at the cell surface. α2/TLR4 binding is associated with TLR4 signal activation, as evidenced by signal inhibition with pHSVsiTLR4 and pHSVsiLA2. However, further studies are needed in order to identify key interacting residues and better elucidate the potential contribution of ligands or scaffold proteins that could alter the three-dimensional protein structure, thereby favoring binding. For example, the assembly of TLR4/TLR6 complexes is regulated by signals from the co-expressed co-receptor CD36 [102], and numerous proteins were implicated as TLR4 ligands. Notable among these are the danger-associated molecular patterns (DAMPs) released from the nucleus and extracellularly as a consequence of injury and inflammation caused by alcohol or any other drug of abuse. They include extracellular matrix molecules (hyaluronan), HMGB1, oxidized low density lipoprotein (oxLDL), oxidized phospholipids (oxPL) or saturated fatty acids, and LPS mimetic ligands of natural origin [90,103,104,105]. However, we have previously shown that the TLR4 signal is innately activated in the non-injured, drug-unexposed P rats [19,20,21,25]. This, together with our current findings that α2 activates the TLR4 signal in untreated, non-injured N2a cells and that the TLR4 signal is also activated in the VTA from naïve (non-drug exposed) P rats, suggests that the potential TLR4 ligand must be a normally expressed intra-cytoplasmic protein. In this context, the cytoplasmic small heat shock protein H11/HspB8, originally cloned in our laboratory [39], is a particularly interesting candidate, because it was shown to be a TLR4 ligand [106] and could function in the activation of the innate TLR4 signal. The use of the proximity ligation assay will extend the capabilities of the traditional co-immunoprecipitation to include ready detection of protein targets, their localization with single molecule resolution, and objective quantification in unmodified cells and tissues [107]. 

Behavioral sensitization to addictive drugs reflects neural adaptations in the dopaminergic mesolimbic pathway that are associated with drug dependence [108]. Nicotine sensitization is a recognized measure of vulnerability to nicotine dependence that complements nicotine self-administration [65]. It manifests as increased locomotor activity as determined by a variety of measures, with the horizontal movement being the most reliable of all activity measures and used in nearly all studies [46,47]. We show that the P rats but not the NP rats are sensitive to the locomotor effects of nicotine and this behavior is blunted by VTA infusion of the pHSVsiTLR4 or pHSVsiLA2 amplicon vectors. Consistent with our previous findings [19,20] these amplicons also blunt binge alcohol drinking, as measured by the operant DIDMSA protocol. The pHSVsiNC amplicon, which does not inhibit the TLR4 signal, although it has identical neurotropic properties, does not blunt the nicotine-mediated locomotor sensitization or binge alcohol drinking, supporting the interpretation that the α2/TLR4 signal regulates alcohol/nicotine co-abuse. We conclude that the amplicons’ effects are through the inhibition of the α2-activated TLR4 signal, because (i) the levels of α2, TLR4, and TH were significantly decreased in the VTA from the P rats infused with pHSVsiLA2 on day 3 post-treatment when both alcohol drinking and nicotine sensitization were maximally reduced, (ii) pHSVsiTLR4 infusion reduced the levels of TLR4 and TH but not α2 (which is upstream of TLR4) on day 3 post-treatment, (iii) the levels of α2, TLR4, and TH in the VTA returned to background levels on day 15 post-treatment when both alcohol drinking and nicotine sensitization were no longer inhibited, consistent with the established duration (10–14 days) of siRNA integrity [19,41], and (iv) behavior was not altered by pHSVsiNC, which does not inhibit the TLR4 signal.

## 5. Conclusions

In conclusion, our data identify α2 as an innate activator of the neuronal TLR4 signal, apparently involving α2/TLR4 binding before exposure to alcohol or nicotine. This activation differs from that mediated by the canonical TLR4 ligand LPS, which is known to function in glial cells, in that it culminates in the activation of CREB, but not NF-kB. CREB is activated through phosphorylated PKA, and the signal results in the upregulation of CRF and TH, both of which are CREB targets, consistent with the role of CRF/CRFR1 in maintaining the activated TLR4 signal [25] and the finding of activated CREB in dopaminergic (TH+) neurons. Compared with the NP rats, the P rats exhibit a unique sensitivity to the locomotor-stimulating effects of nicotine, and both binge alcohol drinking and nicotine sensitization are inhibited by siRNA-mediated knockdown of the α2/TLR4 signal, suggesting that the VTA may have a salient role in the abuse of these two drugs. Significantly, blocking of the α2-regulated TLR4 neuroimmune signal did not affect sucrose drinking; although, palatable food can induce DA release in the nucleus accumbens. This potentially suggests that the downstream contribution of DA, if any, is an important factor in addiction to nicotine [22,23,24] but not in binge alcohol drinking. Moreover, the potential contribution of the brain site and crosstalk between the TLR4^+^ neurons and glial cells, and its role in alcohol-nicotine co-dependence are still unclear. Ongoing studies are designed to address these questions.
